# Segmentation of Potato Consumers Based on Sensory and Attitudinal Aspects †

**DOI:** 10.3390/foods9020161

**Published:** 2020-02-07

**Authors:** Chetan Sharma, Sastry S. Jayanty, Edgar Chambers, Martin Talavera

**Affiliations:** 1 Center for Sensory Analysis and Consumer Behavior, Kansas State University, Olathe, KS 66061, USA; chetansharma@ksu.edu; 2 Department of Horticulture and Landscape Architecture, San Luis Valley Research Center, Colorado State University, Center, CO 81125, USA; Sastry.Jayanty@ColoState.EDU; 3 Center for Sensory Analysis and Consumer Behavior, Kansas State University, Manhattan, NY 66502, USA; eciv@ksu.edu

**Keywords:** potato, preference mapping, consumer, sensory, segmentation

## Abstract

Consumer hedonic scores for potatoes were linked to sensory characteristics to understand the underlying consumer segments, flavor and texture preferences and attitudinal associations regarding potatoes. Consumers were asked to evaluate liking on a 9-point hedonic scale for 12 cultivars of potatoes. Sensory findings were collected by using a consensus-based descriptive analysis approach for the same cultivars. Segmentation analysis procedure identified three subgroups of consumers with different overall liking patterns, indicating variability in the acceptance of different potato cultivars. Drivers of liking were identified for respective segments by using preference mapping. Dissimilar features were found important in determining potato liking patterns. *Purple Majesty*, *Masquerade* and *Rio Colorado* cultivars were found most liked by respondents, while *Russian Banana* were found to be liked the least. Tuber color, price, variety name on package, color of peel, and being locally produced were found to be important factors in purchasing decisions.

## 1. Introduction

Potatoes are among the most consumed vegetables worldwide and among the most versatile and palatable of foods [1]. However, we have recently witnessed a decline in fresh potato consumption [2,3,4,5,6,7,8,9]. Several reasons, such as convenience, long cooking time, flavor, glycemic index, carb-free diets, income, generational changes, and busy lifestyle have been blamed for this decline [5,8,10]. Among these factors, flavor and texture of potatoes were cited as major contributing factors in consumer choice decisions [10,11,12,13]. Several studies have agreed that sensory qualities must be an integral part of trait selection in breeding programs to help slow or stop fresh potato consumption decline [12,14,15]. However, these solitary measures would not be enough to stop declining fresh potato consumption, as consumer purchase decisions do not only involve intrinsic but also extrinsic properties that are not physically part of the product. These are psychographic or attitudinal factors [16]. Attitudinal characteristics are the opinions or beliefs of consumers to diverse topics, in this case, their opinion regarding labeling, pro-social human values, ethics, fair trade features, product origin, sustainability, environment, etc. The rationale behind sensory findings can be better supported with attitudinal measurements, as this helps in making better decisions with respect to each individual’s circumstances [17,18]. In previous studies, consumer choice has been found to be influenced by nutritional knowledge, lifestyle factors, taste preferences, attitudes, and beliefs [5]. The belief that “potatoes are high in carbohydrates” and “starchy materials are not healthy” were found associated with decreased potato consumption among Australian consumers [5]. Psychographic attributes, such as organic and ethical certification, had a positive effect among Italian and German potato consumers with meta-value of openness-to-change, whereas origin was found encouraging attribute for consumers with meta-value of conservation [17]. Another study found that the origin of the potato was a key factor affecting consumer purchasing decisions [19]. In contrast, ‘origin’ was found to not be as significant among British consumers [2]. The development of new and improved (genomic selection for complex traits) potato cultivars that are liked by consumers, and potentially directed at specific market segments, will be of importance to both plant scientists and marketing sections to help increase fresh potato consumption.

Consumer liking can be measured by acceptance tests, and the 9-point hedonic scale has arguably been the most useful sensory method for measuring product liking. In addition to measuring liking ratings to compare acceptance among products, it is often desirable to relate higher/lower liking with other psychographic, sensory and instrumental results to explore and better understand different liking patterns to deliver information that is more thorough and holistic. Preference mapping is a collection of statistical tools used to graphically illustrate the relationships between consumer’s liking and trained panel’s sensory data. Since consumers vary in their liking patterns and purchase decisions depend upon many latent factors, it is often an oversimplification to deliver results based on overall means of consumer data, which may lead to a misrepresentation of the results. Thus, they are often divided into segments that follow distinct patterns that can be attributed to liking, sensory, demographic, and/or psychographic (attitudinal) factors.

This study was designed to discover segments of potato consumers and explore them from a liking, sensory, and attitudinal perspective to help narrow the existing gaps in the understanding of potato flavor and its relation to consumer preferences. Segmentation will help study individual differences among consumers, which can potentially provide a basis for direct marketing aimed at unique market segments [20]. Drivers of liking exploration will further enrich the understanding of consumer choice decisions, key attributes, etc. Furthermore, attitudinal information will empower scientists and marketers with fresh and targeted approaches to better model potato marketing problems. The objective of this study is to assess liking patterns of potatoes and complementing these results with sensory and attitudinal information. We are testing two hypotheses in this study:

**H1.**  *There are different types of potato consumers (segments) based on their liking patterns.*

**H2.**  *These liking patterns can be explained not only by sensory properties but also by some psychographic (attitudinal) characteristics of consumers.*

## 2. Materials and Methods

### 2.1. Sample Preparation and Serving

Potatoes were bred and harvested by the San Luis Valley Research Center at Colorado State University and the Hermiston Agricultural Research and Extension Center at Oregon State University (Table 1). All cultivars were shipped within a week after harvesting. Samples were shipped in burlap bags placed inside carton boxes. Tubers were immediately stored in a dark walk-in refrigerator at 40–41 °F (4–5 °C) and 91–95% humidity upon arrival. Descriptive analysis, which took almost one month for completion from the day samples were received, was conducted initially followed by the consumer study (two-day study), which was conducted after two months with the same sample set. Descriptive and consumer studies were executed at different physical locations, so they were therefore planned and executed separately, as logistics, facilities, scale of operation, and location were different. Fifty-five cultivars of potatoes were used for descriptive analysis, as the goal was to develop sensory language—which required as many samples as possible—whereas only 12 selected cultivars were used for consumer study, as the goal was to understand the role of individual differences in segmentation and role of attitude in obtained segments. For the evaluation, samples were peeled, diced, boiled (for optimum doneness, which was evaluated subjectively by using a fork) and mashed, identified by three-digit codes, and served with no additional condiments in both studies. The serving temperature was the same for both studies (i.e., 145–155 °F).

For the descriptive study, five highly trained panelists from the Center for Sensory Analysis and Consumer Behavior (CSACB), Kansas State University, Manhattan, Kansas, participated in this study. All these panelists had been through 120 h of sensory descriptive analysis panel training with a large variety of food products and have experience evaluating several product categories, including potatoes. A 15-point scale with 0.5 increments was used for intensity quantification of sensory attributes. Diced potatoes were boiled for about 19 ± 3.5 min with respect to cultivar type and subsequently mashed by using handheld ricer (OXO^®^ 3-in-1 Adjustable Potato Ricer, OXO Consumer Care Center Chambersburg, PA, USA). Three tablespoons of boiled potato water was added back into the mashed potato for the purpose of a consistent, smooth texture while being stirred. Sixty to seventy grams of product were served in glass jars (2.3” diameter and 2.1” height) over two hot ceramic tiles covered in aluminum foil to keep the mash potatoes warm during evaluation. Watch glasses were used to cover the sample before evaluation. A paper towel sheet (bounty) was used over hot ceramic tile (two tiles) to prevent sliding. A square steel pan of 8” length × width was used to hold the hot aluminum foil covered ceramic tiles. A ceramic tile of 5.9” length × width was pre-heated in convection oven at 400 °F for about 1 h 30 min. Samples were served (40 g) to panelists within 10 to 15 min holding time. For other details about sample preparation and serving for descriptive analysis, please refer to [3,21]. Cucumber, hot water, and steamed towels were used for palate and nostril cleansing in descriptive analysis.

For the consumer study, potatoes were peeled, diced into cubes and kept immersed in cold water until ready for cooking to minimize browning. When ready, samples were transferred to a jacketed cooking kettle (Cleveland, Model—KET-6-T, Cleveland Range Ltd., Toronto, ON, Canada) and boiled until samples cubes were tender. The cooking temperature for doneness was 175 °F. When fully cooked, samples were drained and mashed using a ricer (Stainless steel potato ricer, 25 oz. capacity, WebstaurantStore, Lancaster, PA, USA). A portion of the boiling water was saved for the final mixing. Once mashed, samples were transferred to the mixer and added 2.5 US cups (40 tablespoons per 15.5 lbs of boiled potato mass) of the saved boiling water until fully mixed (Globe, Model—SP 30, Globe food equipment company, Dayton, OH, USA). Samples were separated in portions based on serving design, packed, vacuum sealed (VacMaster, Model—VP325, 5200 W 110th Suite 200, Overland Park, KS, USA), and kept frozen (5–10 °F) until the day of testing. On the day of testing, a braising pan with tilting skillet (Cleveland, Model—SEL-30-T1, Cleveland Range ltd., Toronto, ON, Canada) was used for thawing samples and a steam table (Duke manufacturing, Model—E304 M, 2305 N Broadway, St. Louis, MO 63102, USA) with counter tops was used for holding warmed samples before serving. Samples were held warm (145 to 155 °F) for about 20 to 25 min. An amount of 42 g/serving was served to consumers. Samples were served plain (no condiments or additional flavors added). More detailed information on sample preparation and serving for consumer study can be found in [3]. The consumer study was conducted under the Institutional Review Board (IRB) approval using approved protocol, and subjects were paid for their participation. Participants signed an informed consent prior to product evaluation. Compusense Cloud (Compusense, Inc., Guelph, ON, Canada) was used for recruitment, screener, and questionnaire preparation as well as data collection. Unsalted crackers were used as a palate cleanser in the consumer study.

### 2.2. Participants Recruitment for Consumer Study

Potato consumers, 18 to 75 years old, from the Kansas City area (*N* = 95; Male = 50, Female = 45) were screened for a two-day study at the Center for Sensory Analysis and Consumer Behavior (CSACB) at Kansas State University, Olathe campus. Consumers were screened to be fresh produce purchaser, frequency of purchase (minimum once a month to weekly), and consumer of potatoes (minimum 1–2 times/week). Compusense cloud (Compusense, Inc., Guelph, ON, Canada) was used for screening and execution data collection.

### 2.3. Questionnaire

Consumers were asked for liking/disliking on a 9-point hedonic scale overall and for appearance, aroma, flavor, and texture at the beginning of the questionnaire. Other questions were also asked to probe consumer behavior with respect to potato purchase decisions. For example, consumers were asked to rank the most important to the least important factors when purchasing potatoes, to rank the most liked to the least liked cooking methods, etc. Varietal information available in the market and its role in purchase decisions was probed by asking consumers “which varieties do you normally purchase? Please check all that apply”. In addition, several agree/disagree statements were asked to assess consumer behavior, the social image of potatoes among consumers and social issues, and sentiments associated with potatoes. All agree or disagree questions were asked using a seven-point Likert scale, with anchors such as “strongly disagree, disagree, somewhat disagree, neither agree nor disagree, somewhat agree, agree, and strongly agree”. Statements were not randomized for agree/disagree questions.

### 2.4. Design of Experiment for Consumer Study

Consumers evaluated six cultivars per day in our study. The design was balanced over the two days where each sample was given once to each subject (monadically) and each sample occurred equal number of times on each position. The crossover design had subject and day as blocks.

### 2.5. Statistical Analysis

Hierarchical cluster analysis (HCA) was used to explore and group consumers based on shared characteristics of overall liking scores for each cultivar type by using JMP Pro 14.3.0 (SAS, Cary, NC, USA). Hierarchical clustering was agglomerative in nature, where each data point formed its own cluster and subsequently merge the next nearest data point until all the data points are in one cluster. The number of cluster(s) were decided by using a distance graph, and Ward’s method of minimum variance was used to measure the distance between cluster(s). The distance was the sum of squares (SS) between two data points summed over all the variables. Null hypothesis (H0) for the experiment was that consumers (or segments) liked all potato cultivars equally, and thus *experimentwise* Type I error rate was of interest to the authors. Controlling the *experimentwise* error rate at α = 0.05 would necessarily control the *comparisonwise* error rate at no more than 0.05. Statistically significant differences in mean liking intensities between segment types were identified by Tukey-Kramer post hoc test. To evaluate the variability between products with respect to consumer liking data, internal preference mapping (IPM) was conducted by using JMP Pro 14.3.0 (SAS, Cary, NC, USA). Principal component analysis was done on covariance matrix by using consumer hedonic data and to explain the differences in liking between samples, sensory (trained panel) data was used as supplementary variables within the hedonic space. Internal preference mapping technique (IPM) was chosen over other techniques, because the analysis is based on consumer hedonic data and trained panel data is supplementary in nature. These are common techniques in marketing and product development. Agree/Disagree scale data was tested for the null hypothesis of no association between opinions/attitudes and consumer segments. The observed frequency of occurrence of “Agree or Disagree” is not associated with the type of segment. Chi-squared test of association was used to test the abovementioned hypothesis statistically.

## 3. Results

### 3.1. Segmentation of Potato Consumers

**Figure 1:**
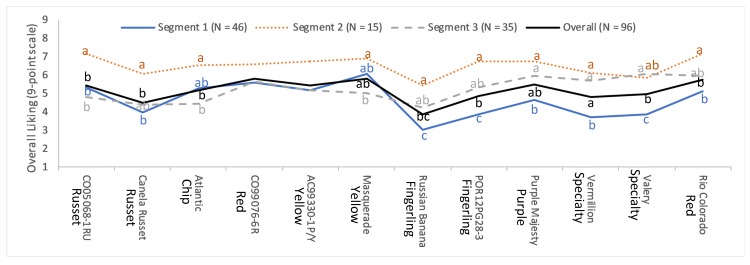
Plot of discovered segments with respect to overall liking score of consumers. Under the null hypothesis of no difference between obtained segments, graphic points with different superscripts implies significant difference within the cultivars (*p* < 0.05). Tukey-Kramer was used as post-hoc analysis for pairwise comparison.

Consumers were grouped into three segments based on their overall liking score (Figure 1). The first segment had almost half (48%) of the total respondents, while the second and third segments had 16% and 36% of respondents, respectively. Significant differences between segments are observed. For example, *Masquerade* was liked by segment one, *CO05068-1RU* and *Rio Colorado* by segment two, *Purple Majesty* and *Rio Colorado* by segment three. However, no such difference was observed between segments for disliking, as all segments disliked Russian Banana. Segments one and three had overall lower scores than segment two.

Color of the flesh may be a discouraging factor for the lower score of segment one respondents, as samples with purple and pink flesh colors received the lowest scores. In addition, segment one showed very narrow choice with respect to overall liking, because the highest liking was reported only towards *Masquerade* cultivar. Contrarily, segment two and three showed a wider range of favorite potato cultivars. Highest overall liking was reported towards *Rio Colorado* (7.13) and *CO05068-1RU* (7.13) in segment two, whereas *Valery* (6.06), *Purple Majesty* (5.97), and *Rio Colorado* (5.97) were reported in segment three, respectively. Overall, segment two respondents had the highest liking scores for all test samples compared to the other segments. *Russian Banana* cultivar was found to be least liked, irrespective of obtained segments. These results support the first hypothesis of this study (H1). Based on the sample of consumers included in this study, there are different types of potato consumers based on their liking patterns.

### 3.2. Preference Mapping for Each Obtained Segment

#### 3.2.1. Internal Preference Mapping—Segment 1

**Figure 2:**
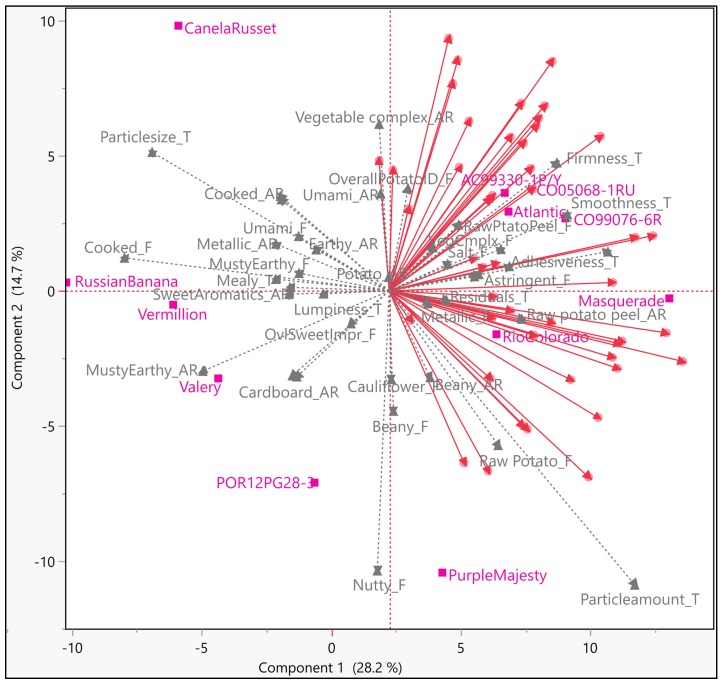
Internal Preference Mapping (IPM) plot for the first segment of respondents (*N* = 46); Magenta colored markers ■ and labels—Samples; gray colored markers ▲ and labels—sensory descriptors; red colored markers ● and vectors—participants/consumers. IPM plots were obtained by using PCA technique on covariance. All sensory descriptors were added as supplementary variables. Homogeneous respondents were pre-identified by using Hierarchical clustering.

The first step towards interpreting IPM was to assess whether a given principal component (PC) or dimension can be adequately approximated by fewer than *p* (original number) variables. About 65% of total variation among samples could be described by using the first four principal components (PCs) (Figure 2). The low accountability of total variation might be due to subtle differences among samples, poor linear relation among variables (consumers), nature of the consumers or other unknown reasons. Vegetable complex, beany, raw potato, raw potato peel, metallic, astringent, cardboard, earthy, salty, overall potato ID, smooth, firm, and adhesive texture seem to be the major drivers of liking for the first segment of respondents, while dominance of cooked, sweet aromatics, cauliflower, musty earthy, grainy, mealy, and texture do not trigger high hedonic ratings (Figure 2). PCs are often interpreted by looking at the loadings for each variable and for this reason, sensory description of the samples could be useful in IPM interpretation. From the size of the coefficients, the first component was dominated by musty-earthy, cooked, earthy, particle size, and particle amount, while the second component was dominated by nutty and particle amount. The third component was dominated by overall potato ID, particle amount, and lumpiness, while the fourth component by cauliflower aroma and earthy aroma. *Russian banana*, *Vermillion*, and *Valery* were found to have a strong intensity of negative drivers of liking.

**Figure 3:**
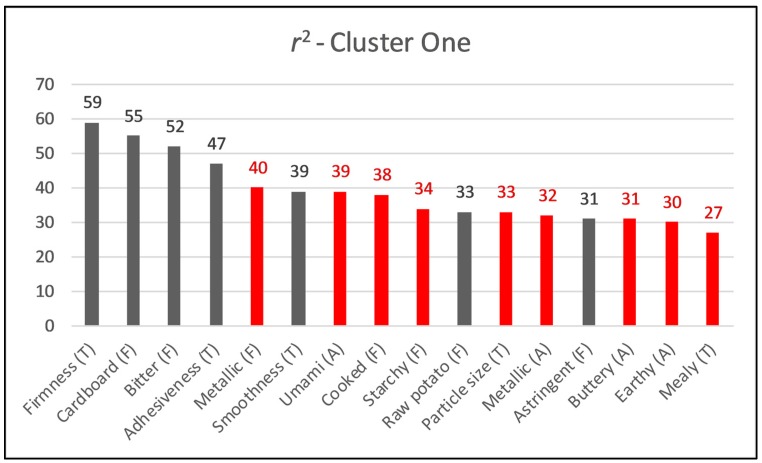
Coefficient of determination (*r*^2^) for the relationship between overall liking and sensory attributes (polynomial equation used for *r*^2^; red bars show negative drivers of liking).

Simple linear regression was performed to complement the obtained results from IPM (Supplemental Figure S1). Average liking score for overall liking was used in function of each attribute, to visualize the positive and negative drivers of liking. Linear and quadratic relationship of attributes were observed with mean liking. From polynomial equation, *r^2^* was obtained and plotted as bar graph in Figure 3. Saturation point phenomenon (pattern where liking starts increasing/decreasing after minimum/maximum point observed) was observed with some attributes such as, potato, earthy, cooked, cardboard, and umami. The cardboard attribute has been mentioned extensively in publications in relation to potato flavor and could qualify as a major flavor character of potato [22,23]. Interestingly, a low level of bitterness was appreciated by consumers, showing a quadratic relation. Similarly, metallic flavor, beany, and vegetable complex aroma also appeared to have quadratic relationships with overall liking.

#### 3.2.2. Internal Preference Mapping—Segment 2

**Figure 4:**
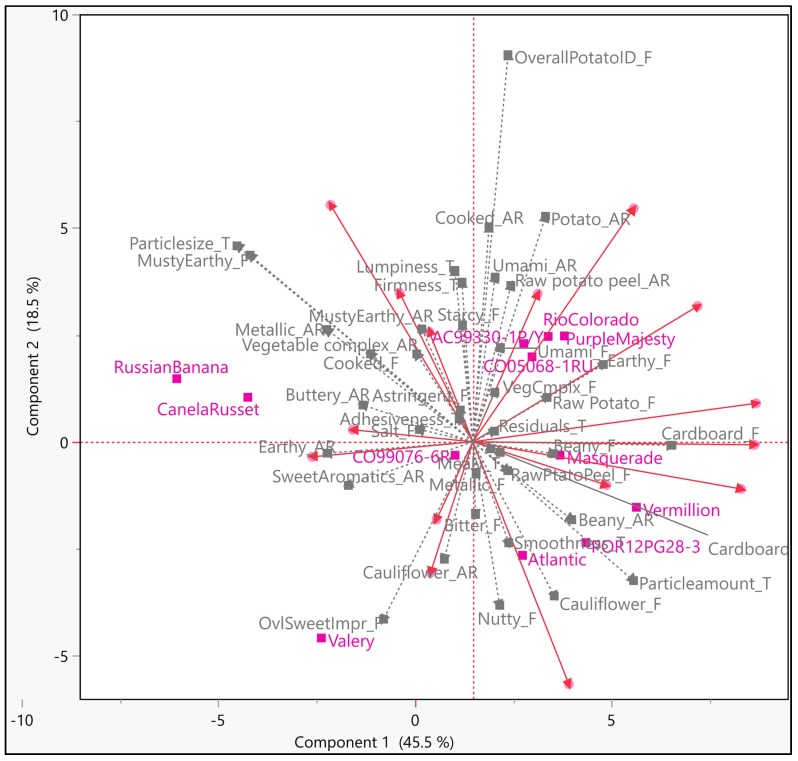
Internal Preference Mapping (IPM) plot for the second segment of respondents (*N* = 15); Magenta colored markers ■ and labels—samples; gray colored markers ▲ and labels—sensory descriptors; red colored markers ● and vectors—participants/consumers.

PCA technique explained higher variability for the second group of respondents (Figure 4). In this cluster, respondents looked more heterogeneous as vectors were found in all directions. Therefore, drivers of liking could not be generalized for this group. Respondents from segment two were found to like all potato types with no particular reason, which could be a reason on itself for a non-aligned vectors direction.

#### 3.2.3. Internal Preference Mapping—Segment 3

**Figure 5:**
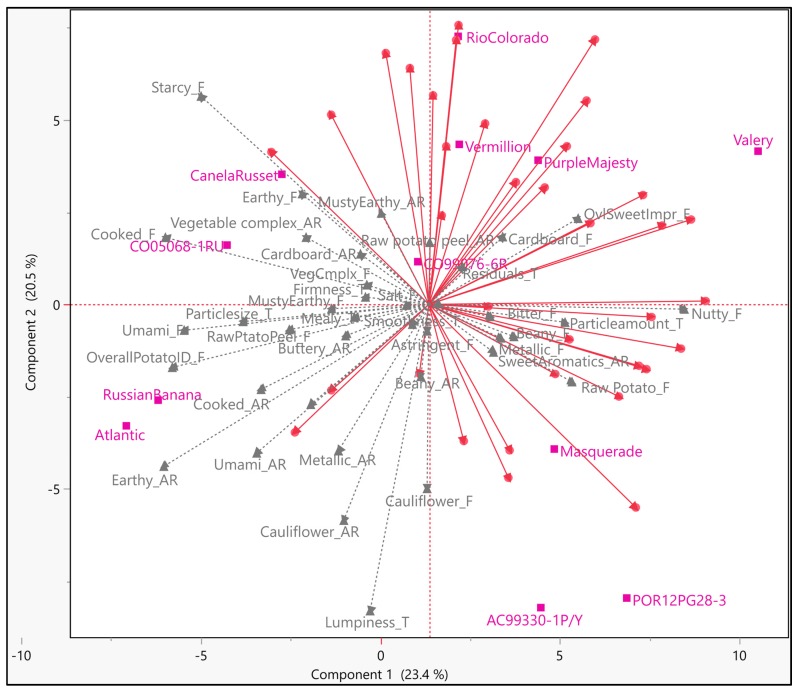
Internal Preference Mapping (IPM) plot for the third segment of respondents (*N* = 35); magenta colored markers ■ and labels—samples; gray colored markers ▲ and labels—sensory descriptors; red colored markers ● and vectors—participants/consumers.

**Figure 6:**
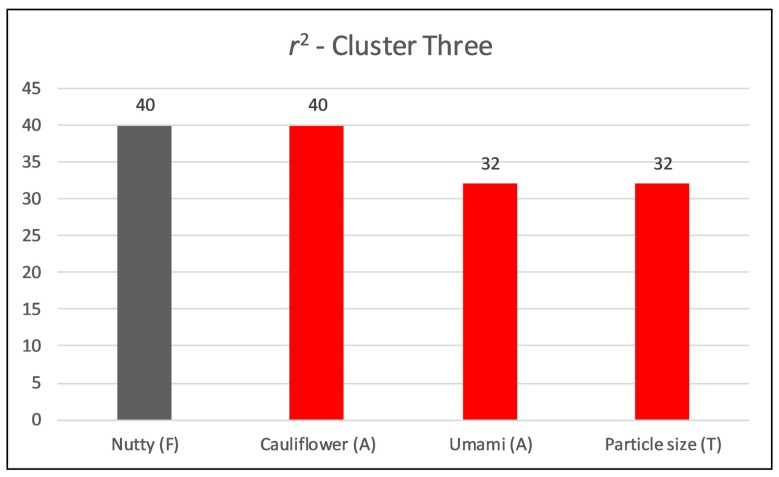
Coefficient of determination (*r*^2^) for the relationship between overall liking and sensory attributes (polynomial equation used for *r*^2^; red bars show negative drivers of liking).

Respondents in group three looked reasonably homogeneous, making generalization of the drivers more robust for this group (Figure 5). The first three components explained around 60% of total variation. Overall sweet impression, sweet aromatics, beany, nutty, cardboard, bitter, raw potato, and cauliflower flavors appeared to trigger hedonic ratings for this group of respondents (Figure 5). From a texture standpoint, particle amount and residuals appeared to prompt hedonic ratings. Compared to segment one, this group of respondents had a higher liking for toasted, nutty, and sweet impression. The first component was found to be dominated by earthy aroma, overall potato ID, starchy, umami, cooked, and nutty flavors, while the second component was found dominated by cauliflower aroma, starchy flavor, and lumpy texture. The third component was found dominated by musty-earthy, cooked and raw potato peel aromas, as raw potato, cooked, earthy and musty-earthy flavors. About 40% of variation in overall liking can be explained by nutty flavor (Figure 6). Several quadratic relations were observed with attributes, such as, nutty, cauliflower, etc. (Supplemental Figure S2).

### 3.3. Factors Affecting Purchase Decisions

Overall, price was found being the most important factor (25%) during purchase, followed by variety (16%), color of peel and size (15%), being locally produced (11%), nutritional information (9%), shape (5%) packaging, and organic (2%). Organic was found being the least important factor for consumers (31%), followed by packaging (26%), nutritional information (18%), locally produced (6%), variety, color of peel and size (4%), price, and shape (3%). Compared to proportions, median value was calculated to find the central tendency for ranked data and no difference was found between size and price rank (Table 2). Similarly, shape and variety were found sharing rank five for the least important factors. Some gender differences were observed, as males rated shape (54% vs. 36%), organic (20% vs. 6%), and locally produced (34% vs. 24%) compared to female consumers, whereas price was rated higher by female consumers (88% vs. 70%). Similarly, a difference in least important factors among males and females was found, such as shape and price, which were found to be the least important factors for males compared to females (20% vs. 6% and 16% vs. 6%, respectively). Nutritional information (67% vs. 60%), organic (85% vs. 72%) and locally produced (65% vs. 58%) were found to be the least important for female consumers compared to male consumers. The choice of important factors was also found to be affected with household income groups, such as size of potato and color of peel, which were not as important factors for low household income groups compared to high household income groups. Being locally produced was found to not be an important factor for high household income groups. Importance of locally produced and color of peel factors was found depleting with the increasing education level.

### 3.4. Consumer Choice of Cooking Methods

Baked and mashed potato cooking methods were ranked as the most liked potato preparation methods followed by fried, boiled, and microwaved (Table 3). From a proportion standpoint, mashed method of potato preparation was cited as the most liked (44%), followed by baked (28%). Microwaved method was the least liked method (19%) of cooking potatoes, followed by boiling (11%).

### 3.5. Potato Variety Preference

**Figure 7:**
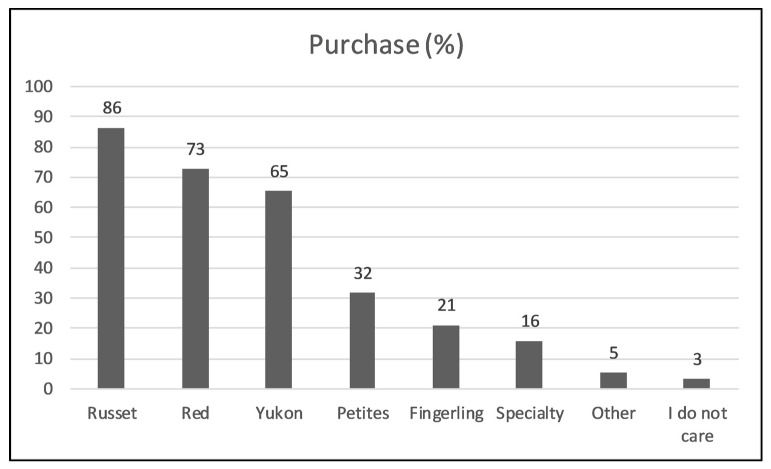
Consumer’s purchase percentage for different varieties (*N* = 95). ***Question***—Which potato varieties do you normally purchase? Please check all that apply.

Russets, red and Yukon were found to be the most frequently checked varieties by consumers (Figure 7). Only 3% of consumers stated that they do not care about varieties when making a purchase, indicating the growing importance of variety name on the package.

### 3.6. Attitudes, Opinions and Perceptions

**Figure 8:**
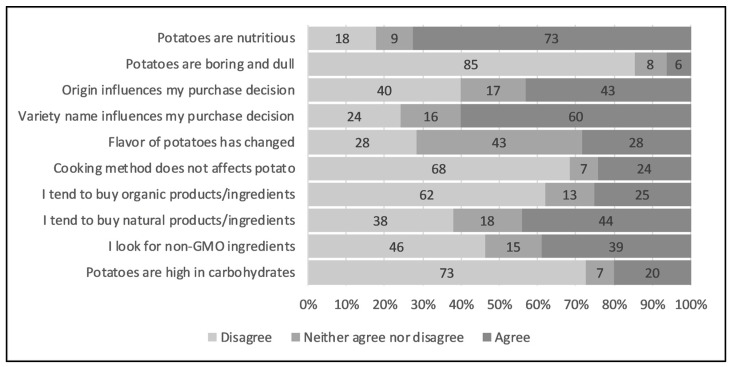
Belief and attitudes inquiring of Kansas City respondents (*N* = 95).

Consumers were asked to indicate the extent to which they agreed or disagreed with several statements about potatoes (Figure 8). Segments were tested for their association with the attitudinal statements by using the χ^2^ test of association. No significant association between the opinion held by a sample of consumers and segments (based on overall liking) was found for most of the attitudinal statements. Psychographic (attitudinal) attributes affect consumers’ purchase behavior [16] but not necessarily blind sample liking, and since segments were obtained based on the overall liking, the observed disconnect was expected. Still, we can observe some directional associations that partially support the second hypothesis (H2) that certain psychographic characteristics, in addition to sensory properties, can help differentiate these segments (Supplemental Figure S3). Consumer associations with attitudinal characteristics generate sensory expectations that can affect perception and hedonic ratings [16]. However, no such information was provided to the participants (samples were served blind). Nevertheless, flavor certainly affects choice, but trade-offs regarding convenience, origin, brand, etc., at purchase points, and ultimately decides the fate of the product.

Table 4 shows summarized information and overall assessment for each segment including hedonic, sensory, and psychographic (attitudinal) aspects.

- Potatoes are nutritious (*N* = 95). Potatoes were found to be nutritious by 73% of total consumers, whereas 18% of consumers were found to have disagreement with this statement.
- Potatoes are boring and dull (*N* = 95). Only 5% of consumers agreed with this statement.
- Labeling about origin (such as Idaho, Colorado etc.) of potatoes influence my purchase decision (*N* = 95). Two distinct groups were observed with respect to this statement, as 44% respondents agreed whereas 40% disagreed.
- Labeling about variety name (such as Russet Norkotah, Snowden etc.) on the package influences my purchase decision (*N* = 95). A significant effect of variety name was observed on purchase decision, as 61% of respondents agreed with this statement while 24% disagreed.
- I believe that the flavor of potatoes has changed historically (*N* = 95). Respondents were found having no clear trend about this statement, as 29% agreed, whereas 28% disagreed. The highest percentage of the ‘neither agree nor disagree’ option was chosen by the respondents (43%) for the abovementioned statement, implying the lack of belief or relevance (understanding) of this statement.
- Potatoes are healthy regardless of the cooking method (*N* = 95). Overall, 68% of respondents disagreed with this statement while 24% agreed.
- I tend to buy organic products/ingredients (*N* = 95). Overall, 62% respondents disagreed with this statement while 23% agreed. Gender’s attitude differences with respect to food safety and environmental contamination could be considered by this statement, as female respondents were found less attentive towards these issues (67% were disagreed) compared to male respondents (58% disagreed).
- I tend to buy natural products or ingredients (*N* = 95). Natural product interest would indirectly measure the organic statement mentioned above. Overall, 38% respondents disagreed with this statement while 44% agreed. A higher percentage of female respondents compared to male respondents (22 vs. 14%) were found to have uncertainty about whether the product being organic would affect their purchase decision or not, as they selected ‘neither agree nor disagree’. Male respondents agreed with the statement significantly higher compared to female respondents (50 vs. 38%).
- I look for non-GMO (Genetically Modified Organisms) ingredients in the food I eat (*N* = 95). Higher proportion of respondents (46%) disagreed with the statement that they look for non-GMO ingredients in their food compared to those who agreed (38%). Male respondents disagreed with this statement significantly more than female respondents (52 vs. 40%, respectively).
- I avoid potatoes because they are high in carbohydrates (*N* = 95). Overall, 73% respondents disagreed while 20% agreed with the statement. Most of the respondents, irrespective of gender differences, were found to disagree (73% female vs. 72% male) with this statement.

## 4. Discussion

### 4.1. Segmentation of Potato Consumers

This study supports the first hypotheses (H1) that there are different types of potato consumers based on their liking patterns. Each segment was also investigated for their relationship with household income and level of education. No major differences were observed within those demographic parameters. The reason for this could be that potatoes are not a premium product, but rather a more mainstream product enjoyed by all segments of the population, regardless of income and educational differences. However, the level of education was previously found to have a positive effect on the consumption of organic potatoes [23]. Attitudinal dissimilarities between segments were investigated by agree/disagree questions (Table 4). This study partially supports H2 as some directional differences are observed. Varietal information on the package affects segment one respondents purchase decisions. Compared to varietal information, the origin of the potato was not as important for segment one respondents as it was for segment three respondents. Segment one respondents appeared to be consumers of more conventional potatoes and were less interested about natural, organic, non-GMO, and carbohydrates content. More aisle options might confuse these respondents and thus they are focused on only potatoes. Segment two respondents had a higher liking for practically all samples, so this segment was called the ‘potato lovers’ group’. Respondents in this segment tended to disagree (33%) with the statement that potatoes are nutritious compared to the other two segments. It was somewhat expected that segment two respondents would have lower agreement with this statement since they eat for pleasure (segment two respondents were in complete disagreement with the statement “potatoes are boring and dull”). For segment two respondents, labeling about origin seemed to have some influence on purchase decisions. These respondents, who reported that origin was more instrumental over variety, appeared to have some idea about potato flavor, as they reported the highest agreement with the statement that the “flavor of potatoes has changed historically”. Belief about origin may share some cognitive concept with flavor, which could lead respondents to believe that flavor of potatoes has changed historically. Organic ingredients appear more liked by segment two respondents, strengthening the construct where origin, historic variation of flavor, and organic production have higher importance. Segment two consumers also had the highest agreement with the statement that they avoid potatoes because of a high carbohydrate content and that they look for non-GMO content. In summary, segment two appears to be more characterized for being natural and organic seekers, non-GMO, give importance to geographical origin and are more nutritionally informed. However, this was the smallest segment (*N* = 15). With respect to package and label requirements, segment three agreed with the statement that labeling about origin influence their purchase decision. Similar findings were reported previously, where health-oriented participants quote origin, local, health and safety of the food over price [24]. The third segment was found to be more open to “alternative” varieties of potatoes such as those with purple or pink skin and interior. Therefore, it was assumed that for this segment, labeling about variety name on the package would certainly influence their purchase decision, which was supported by the results. Therefore, the third segment pays more importance to package information. Consumers in segment three seem to want more information on the package including variety name and origin, more natural than organic and least agree with the statement that flavor has changed historically. A previous study found that health-oriented consumers were mostly females, which were highly educated, had higher income, and more children compared to price-oriented segment [23]. Overall, the word ‘natural’ was more influencing than ‘organic’, irrespective of segments.

### 4.2. Factors Influencing Purchase Decisions

Contrary to [24] findings of lower importance of price by Maine respondents, price was found highly important in our study (conducted in the Kansas City (KC) area). Being a cash crop (produced for its commercial value rather than for use by the grower), potato size has huge economic importance, and even the size of potato packs has been documented previously as an important factor [19]. Gareau (2018) found the big size of potatoes a reason for consumers’ turning away from it. Appearance (79%), flavor (72%), size (59%), nutrition (57%), texture (55%), and price (48%) were found to be the most important attributes for Colorado consumers, whereas variety and organic certification were the least important [25]. Massachusetts consumers were found to be favorable towards the “Maine” label, whereas North Carolina consumers towards “Idaho” label [19]. Brand label effect was found missing in most studies [19]. Being locally produced, firmness, and transparent packaging were found to be the most influential factors in making fresh potato purchase decision [11].

### 4.3. Potato Cooking Method and Convenience

Baking and mashing were reported as most the liked potato preparation methods in this study, excluding convenience and time constraints, which could otherwise affect these results. Demand for processed potatoes is increasing in both domestic and international markets. Boiling has been previously cited as the most common method of potato preparation worldwide, but the demand of instant mashed potatoes has been cited increasingly [25]. In the 1990s, the baking method (47%) was found to be a favorite among Washington state consumers, followed by mashing (30%) and French fries (16%) [26]. Similarly, baking (69%) was found to be the preferred method of fresh potato preparation in Maine consumers, followed by mashing (59%) and roasting (41%) [24]. Women expressed a slightly higher interest in baked potatoes while men expressed a greater interest in roasted potatoes. Mashed potatoes were the preferred choice for respondents of 20 to 50 years of age while baked potatoes were preferred for respondents older than 50 years of age [24].

### 4.4. Potato Variety Preference

This study showed that varieties normally purchased by consumers around the KC area are Russet, followed by Red and Yukon. *Yukon Gold* has been previously stated as the most important variety by Colorado consumers, followed by *Russet Burbank* and *Russet Nugget* [25]. That study reported no variety preference for about 55% of Colorado consumers [25]. Compared to Colorado consumers, Russets were the most popular (55%) among Washington state consumers, followed by White (18%) and Red (9%) potatoes [26]. A high percentage of no preference consumers (18%) was also observed at that time. About 50% of consumers were found having no favorite variety in the Delaware region, whereas 27% selected the Red variety, followed by Russets (13.6%), White (4.3%), *Yukon Gold* (3.1%) and Yellow (1.5%) [27]. Another study found variety of potato factor not significant in purchase making decisions [11]. The difference in question structure among different studies could possibly have some influence on the obtained results, as in some cases respondents were prompted to write the name of the variety, whereas in other cases they had to select the variety from an existing list.

### 4.5. Beliefs, Attitudes, Opinions, and Perceptions

Over 96% of Maine respondents considered potatoes as a healthy food compared to 73% of Kansas City respondents in our study. Maine, as a potato growing state could have a healthy perception of potatoes due to the long history of potato in the state and better marketing strategies. A healthy perception of potatoes aligns with previous findings [10,11,24]. The perception of “potatoes are boring” was found to be higher (<20% agreed) among British consumers [2] compared to US consumers (<10% agreed). In another study, only 5% Australian consumers stated that potatoes are boring [5]. The perception of being boring and/or old fashioned has been found to not be an issue to most potato consumers [5]. Consumer’s positive perception about origin or source stamp on package has been documented extensively [19,24,25,27,28]. Overall, low positive perception of the potato source label among Kansas City respondents could be explained by the fact that Kansas is a non-potato growing state and respondents usually favor local produce [29]. Gender and having a college degree were previously found having little impact on a shopper’s decision to buy organic produce [30]. Consumers’ perception and opinion about “what constitutes natural” was questioned previously and found that chemical sounding names and the age of the consumers influence whether an ingredient or food would be considered “natural” [31]. The impact of “organic” in purchase decisions was found not important for most consumers in our study. This is similar to another study that also reported “organic” as a less important factor in purchase decisions [24]. Demand for organic products was found to differ geographically, as consumers from California and East coast were found more open to organic products than the more traditional Midwest and South [24]. The perception of ‘organic’ was found associated with being healthy, having a trustable origin, and safety, among Germans [23]. Female participants have been found to be strong advocators of ‘organic’ [23]. Consumer understanding of GMOs was found low among US consumers, with just 48% knowing that GMOs were available in market and 16% knowing nothing at all [32]. Perceptions about potatoes such as “High in carbohydrates” and “Starchy vegetables are not healthy” have been previously found to be a reason for the decline in potato consumption [5]. Nutritional value and flavor appeared as the most important driving forces for future potato clone’s improvement [5,25].

## 5. Limitations of This Study

Only a single indicator was used to capture consumer’s attitudes to an issue in agree/disagree questions, which could be limitation as more indicators (to explore attitudinal constructs, nutritional and health awareness, price, etc.) would produce better differentiation and breadth of the information. A relatively small sample size is another limitation because it resulted in small clusters and few statistical differences, especially for the attitudinal portion, making the results of this study directional in nature. A larger sample size could generate more statistical differences and a more representative sample of different areas of the country (as this study only included consumers from the KC area). Logistics, time constraints, and feasibility (such as a two-day study) one preparation method (mashed), consumer fatigue, etc. were other limitations that should be considered.

## 6. Conclusions

H1 is supported by this study and H2 is partially supported due to a small sample size and few significant differences in the attitudinal portion. Three segments of potato consumers were found in this study, characterized by: (1) a price-oriented, conventional potato group; (2) a potato lovers’ group; and (3) a health-oriented, new variety seeker group. Vegetable complex, beany, raw potato, raw potato peel, metallic, astringent, cardboard, earthy, salty, overall potato ID, smooth, firm, and an adhesive texture seem to be the major drivers of liking for the first segment of respondents. Segment two respondents were few in number and their drivers were not aligned to one direction. Overall sweet impression, sweet aromatics, beany, nutty, cardboard, bitter, raw potato, and cauliflower flavors appeared to be triggering hedonic ratings for the third group of respondents. Most respondents considered potatoes healthy; thus, marketing of potatoes should be built around this opinion. Most KC consumers liked the mashing preparation method as a preferred cooking method for potatoes. Kansas state consumers belong to a non-potato producing state, so obtained results can be generalized and compared with other consumers from non-potato producing states for segments, drivers of liking, and psychographic (attitudinal) attributes. The practical implications of this study will be important to breeders, growers, sensory science, marketers, and consumers.

## Figures and Tables

**Table 1:** Potato cultivars used in the study.

S. No.	Clone	Type	Origin
1	CO05068-1RU	Russet	Colorado
2	Canela Russet	Russet	Colorado
3	Atlantic	Chip	Oregon
4	CO99076-6R	Red	Colorado
5	Rio Colorado	Red	Colorado
6	AC99330-1P/Y	Yellow	Colorado
7	Masquerade	Yellow	Colorado
8	Russian Banana	Fingerling	Oregon
9	POR12PG28-3	Fingerling	Oregon
10	Purple Majesty	Purple	Oregon
11	Vermillion	Specialty	Oregon
12	Valery	Specialty	Oregon

**Table 2:** Factors influencing purchase decision (*N* = 95).

Factors Influencing Purchase Decision	Median	Importance
Size	3	Highly important
Price	3	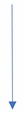
Color of Peel	4	
Shape	5	
Variety	5	
Locally produced	6	
Packaging	7	
Nutritional Information	7	
Organic	8	Least important

Question—Please rank the items in order from most important to least important to you when purchasing potatoes. Click and drag the MOST IMPORTANT FACTOR for you into the 1st box. Click and drag the LEAST IMPORTANT FACTOR for you into the 9th box.

**Table 3:** Favorite potato preparation method

Potato Preparation Method	Median	Importance
Baked	2	Highly important
Mashed	2	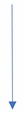
Fried	3	
Boiled	4	
Microwaved	5	
Other	6	Least important

Question—Rank in order the potato cooking method you like most (to least) that you, yourself like to eat. Click and drag the name of the method you LIKED THE MOST into the 1st box. Click and drag the name of the method you LIKED THE LEAST into the 6th box.

**Table 4:** Sensory and attitudinal properties of obtained segments.

	Segment One (48%)	Segment Two (16%)	Segment Three (36%)
Sensory/hedonic aspects	Overall, lowest liking for some samples	Overall, all of the tested samples were highly liked	Intermediate liking
	Very narrow choice	Broad choice	Broad choice
	Highest liking (6.06) was reported for *Masquerade*	Highest liking (7.13) was reported for *CO05068-1RU* and *Rio Colorado*	Highest liking (6.06 and 5.97) was reported for *Valery* and *Purple Majesty, Rio Colorado*
	*Russian Banana* was least liked	*Russian Banana* was least liked	*Russian Banana* was least liked
	Lower hedonic score for colored varieties	Not much impact of color	Higher hedonic score for colored varieties
	Raw potato, earthy, cardboard, vegetable complex and typical potato flavor are key flavor attributes	No specific attributes are associated with this segment	Sweet aromatics, nutty, and bitter are key flavor attributes
Attitudinal aspects	Nutritional information matters	Nutritional information does not matter as much	Nutritional information matters
	Some boredom associated with potatoes	No boredom	Some boredom associated with potatoes
	Origin does not matter as much	Origin matters	Origin matters
	Variety name matters	Variety name does not matter as much	Variety name matters
	Package information is important	No impact of package information	Package information is highly important
	Potato flavor has not changed much historically	Believes that potato flavor has changed historically	Potato flavor has not changed much historically
	Cooking method affects potato nutrition	Cooking method does not impact potato nutrition	Cooking method affects potato nutrition
	Organic, natural are least important	Organic, natural are important	Organic, natural are important
	Not as much opposition to GMO	Non-GMO supporter	Not as much opposition to GMO
Consumer Segments	Price oriented, conventional potato user group	Blind potato lovers’ group	Health oriented, acceptor of new color varieties group

## Supplementary Materials

The following are available online at https://www.mdpi.com/2304-8158/9/2/161/s1, Figure S1: IPM trace plots of each attribute with respect to consumers in cluster 1 OVERALL LIKING for potato samples; grey dotted line—Linear equation, red dotted line—Polynomial equation, Figure S2: IPM trace plots of each attribute with respect to consumers in cluster 3 OVERALL LIKING for potato samples; grey dotted line—Linear equation, red dotted line—Polynomial equation, Figure S3: Relation of opinion to obtained segments.
