# Supplementary material for: Segmentation of Potato Consumers Based on Sensory and Attitudinal Aspects
Source: Foods. 2020 Feb 7;9(2):161. doi: 10.3390/foods9020161 (PMC7073660; doi:10.3390/foods9020161)
Supplement: Supplementary file 1 [file foods-09-00161-s001.pdf]

Segmentation of potato consumers based on sensory and attitudinal aspects

Chetan Sharma[1], Sastry Jayanty[2], Edgar Chambers IV[3] and Martin Talavera[1]

Corresponding author: Dr. Martin Talavera, Email: talavera@ksu.edu, Phone: 913 307 732

[1] Centre for Sensory Analysis and Consumer Behavior, Kansas State University, Olathe – 66061
[2] San Luis Valley Research Center, Department of Horticulture and Landscape Architecture, Colorado State University, Center – 81125
[3] Centre for Sensory Analysis and Consumer Behavior, Kansas State University, Manhattan – 66502

Supplementary data files –

Fig. 1 IPM trace plots of each attribute with respect to consumers in <u>cluster 1</u> <u>OVERALL LIKING</u> for potato samples; grey dotted line – Linear equation, red dotted line – Polynomial equation

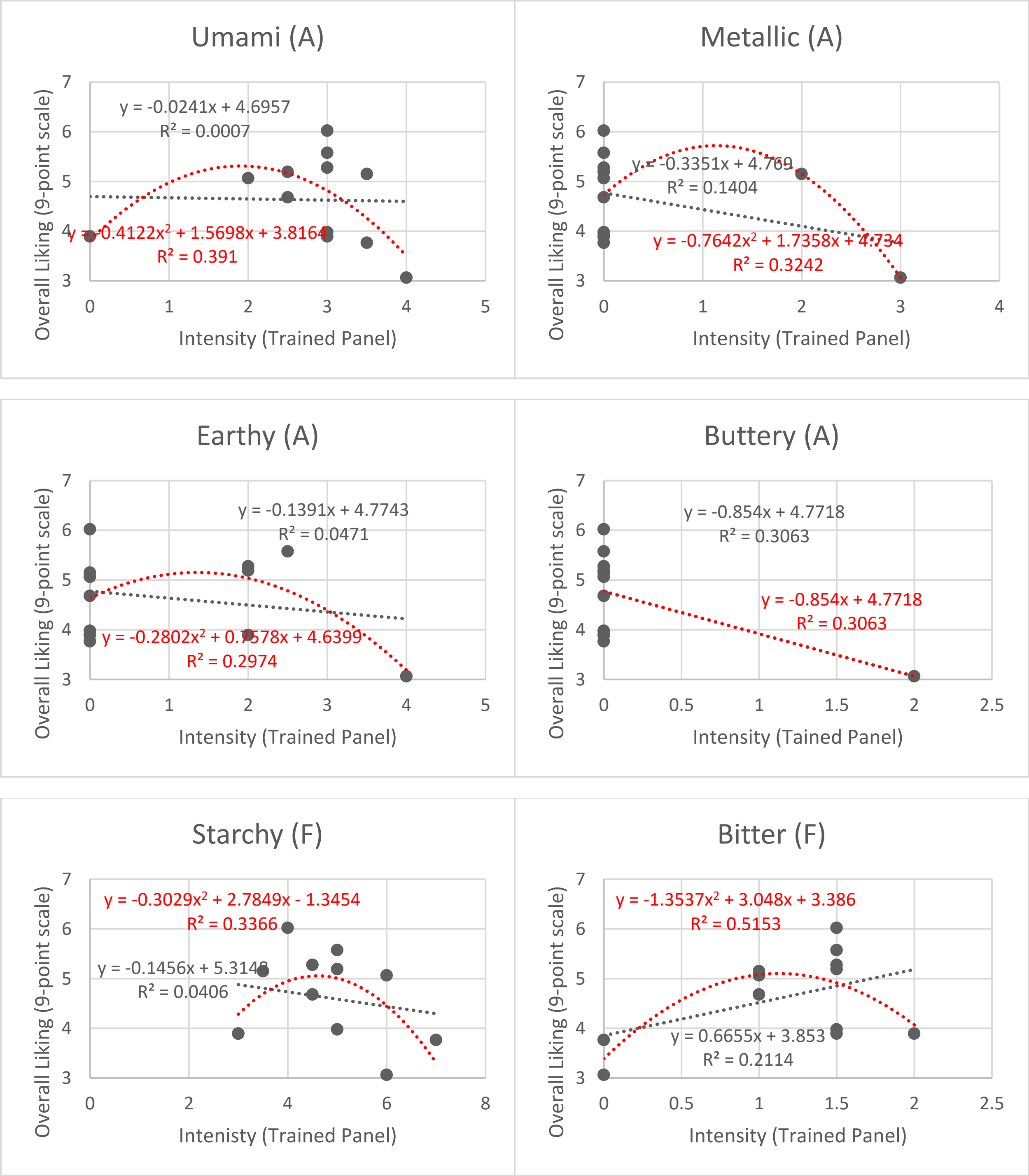

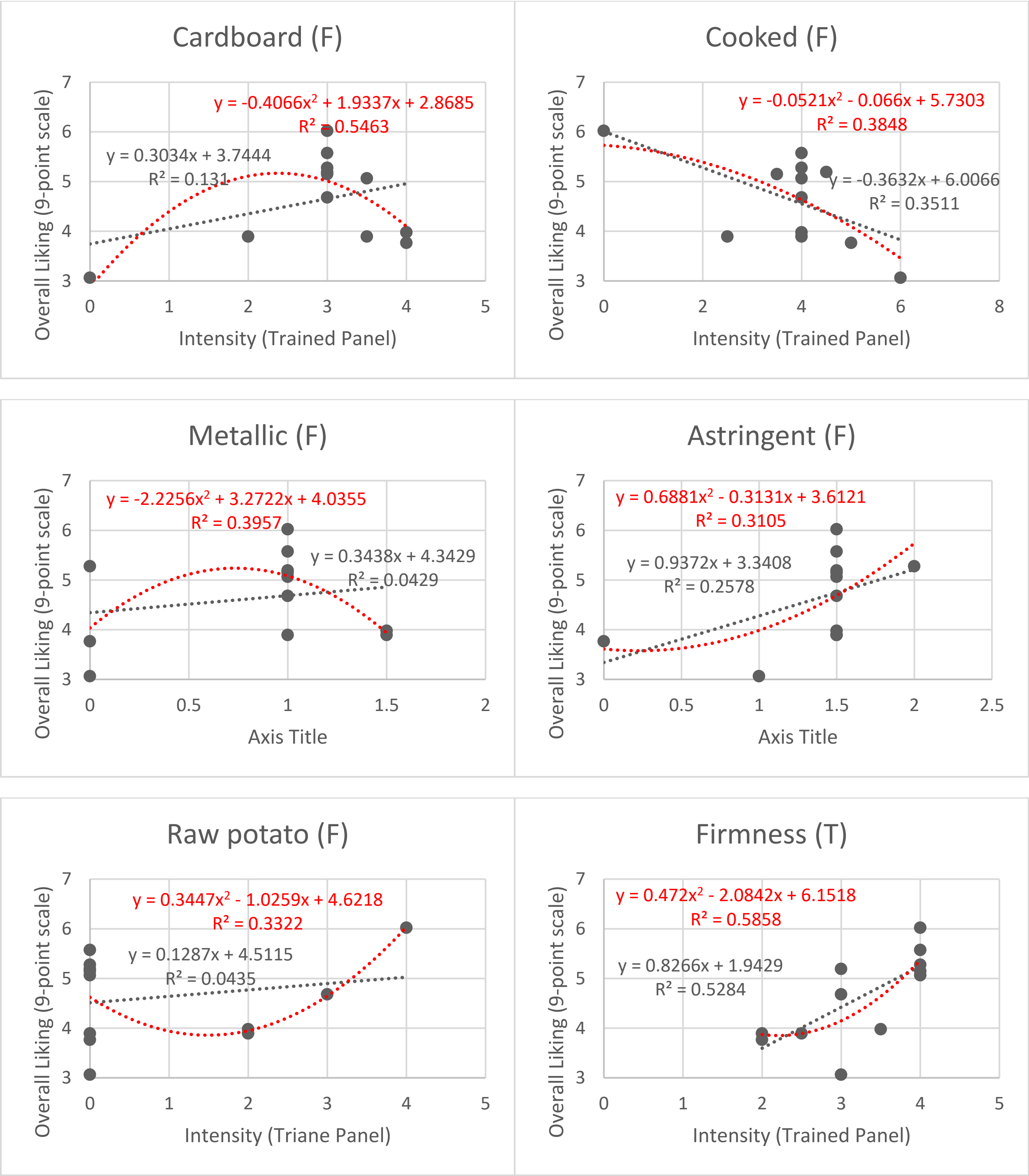
Cardboard (F)
y = -0.4066x² + 1.9337x + 2.8685
R² = 0.5463
y = 0.3034x + 3.7444
R² = 0.131
Overall Liking (9-point scale)
Intensity (Trained Panel)
Cooked (F)
y = -0.0521x² - 0.066x + 5.7303
R² = 0.3848
y = -0.3632x + 6.0066
R² = 0.3511
Overall Liking (9-point scale)
Intensity (Trained Panel)
Metallic (F)
y = -2.2256x² + 3.2722x + 4.0355
R² = 0.3957
y = 0.3438x + 4.3429
R² = 0.0429
Overall Liking (9-point scale)
Axis Title
Astringent (F)
y = 0.6881x² - 0.3131x + 3.6121
R² = 0.3105
y = 0.9372x + 3.3408
R² = 0.2578
Overall Liking (9-point scale)
Axis Title
Raw potato (F)
y = 0.3447x² - 1.0259x + 4.6218
R² = 0.3322
y = 0.1287x + 4.5115
R² = 0.0435
Overall Liking (9-point scale)
Intensity (Triane Panel)
Firmness (T)
y = 0.472x² - 2.0842x + 6.1518
R² = 0.5858
y = 0.8266x + 1.9429
R² = 0.5284
Overall Liking (9-point scale)
Intensity (Trained Panel)

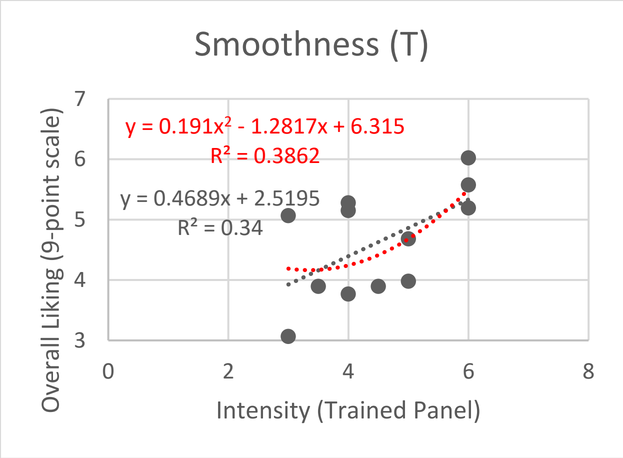
Smoothness (T)
y = 0.191x² - 1.2817x + 6.315
R² = 0.3862
y = 0.4689x + 2.5195
R² = 0.34
Overall Liking (9-point scale)
Intensity (Trained Panel)

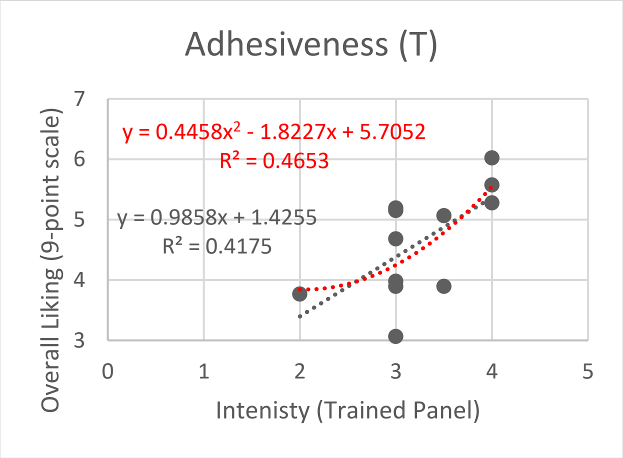
Adhesiveness (T)
y = 0.4458x² - 1.8227x + 5.7052
R² = 0.4653
y = 0.9858x + 1.4255
R² = 0.4175
Overall Liking (9-point scale)
Intenisty (Trained Panel)

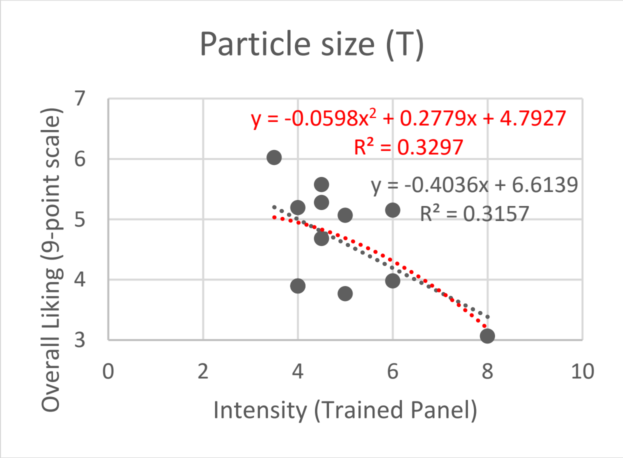
Particle size (T)
y = -0.0598x² + 0.2779x + 4.7927
R² = 0.3297
y = -0.4036x + 6.6139
R² = 0.3157
Overall Liking (9-point scale)
Intensity (Trained Panel)

Fig. 2 IPM trace plots of each attribute with respect to consumers in cluster 3 OVERALL LIKING for potato samples; grey dotted line – Linear equation, red dotted line – Polynomial equation

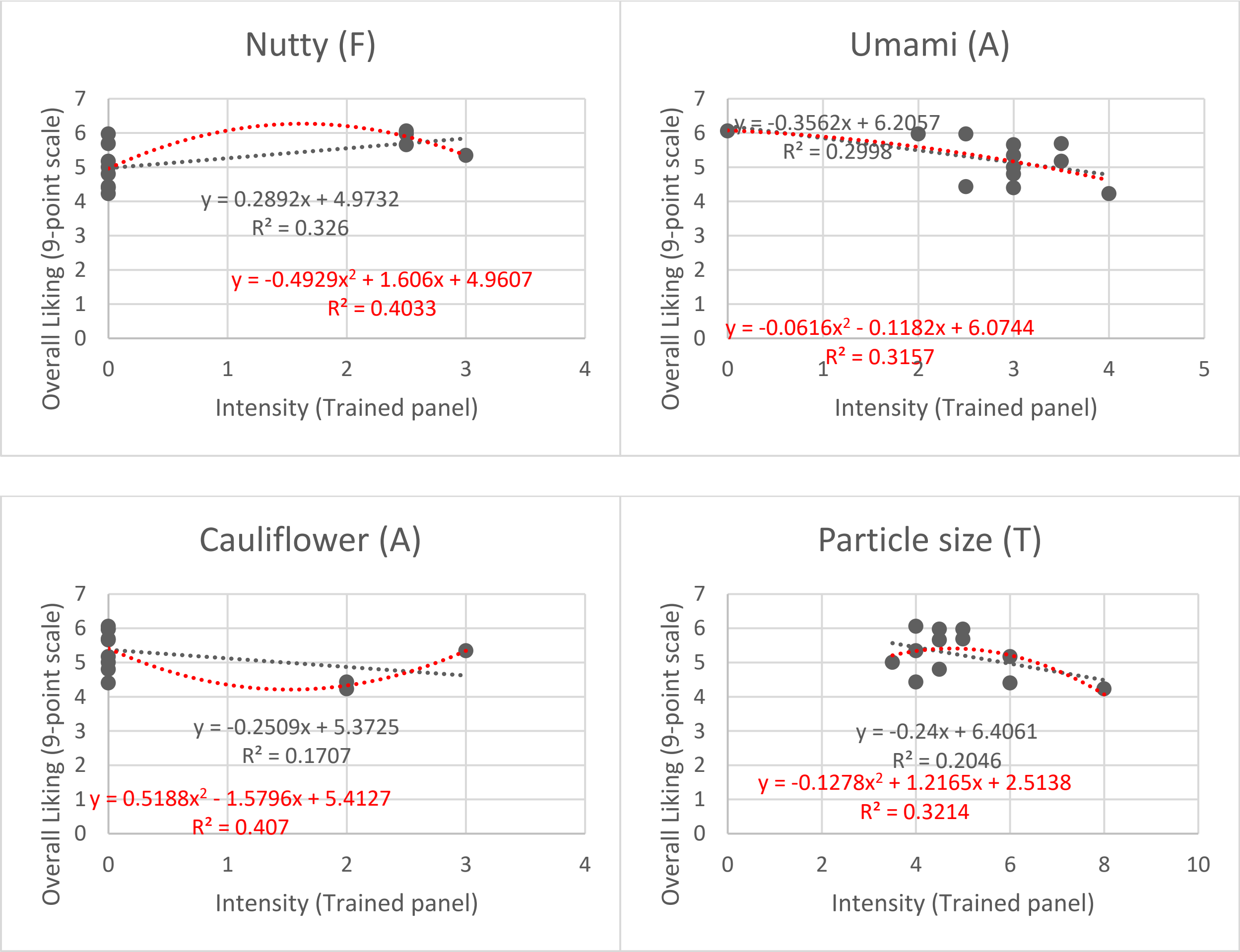

Fig. 3 Relation of opinion to obtained segments[4]

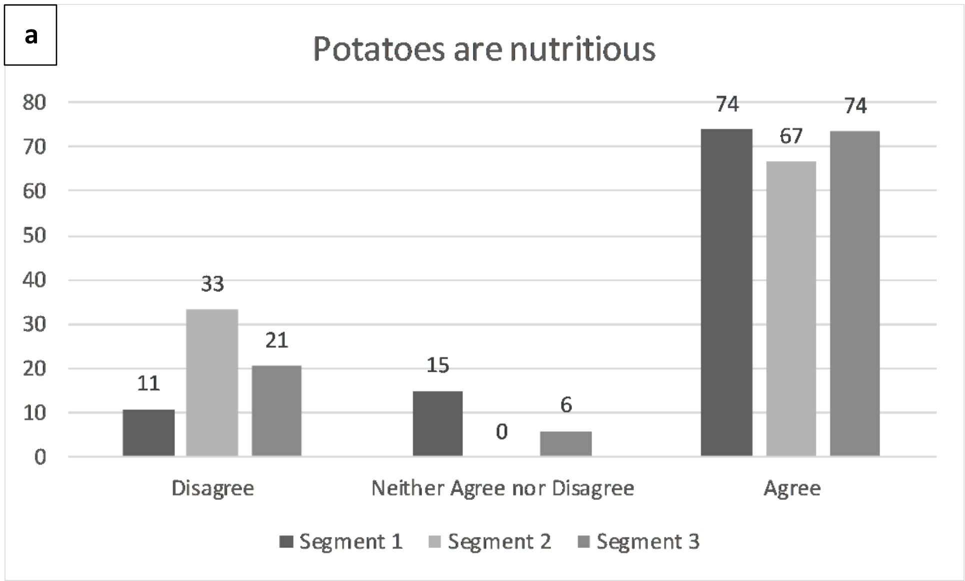

Opinion – Potatoes are nutritious♦ (*N* = 95)

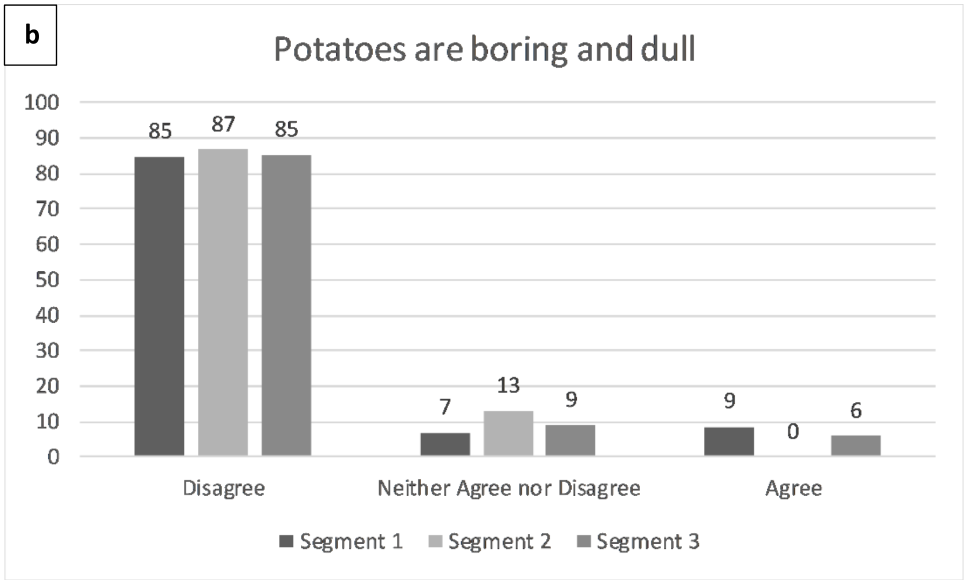

Opinion – Potatoes are boring and dull♦ (*N* = 95)

[4] Under the null hypothesis of no association between the held by a sample of people with their segments opinion (a to j, respectively). χ^2 test of association was used to test the hypothesis.

♦ not significant

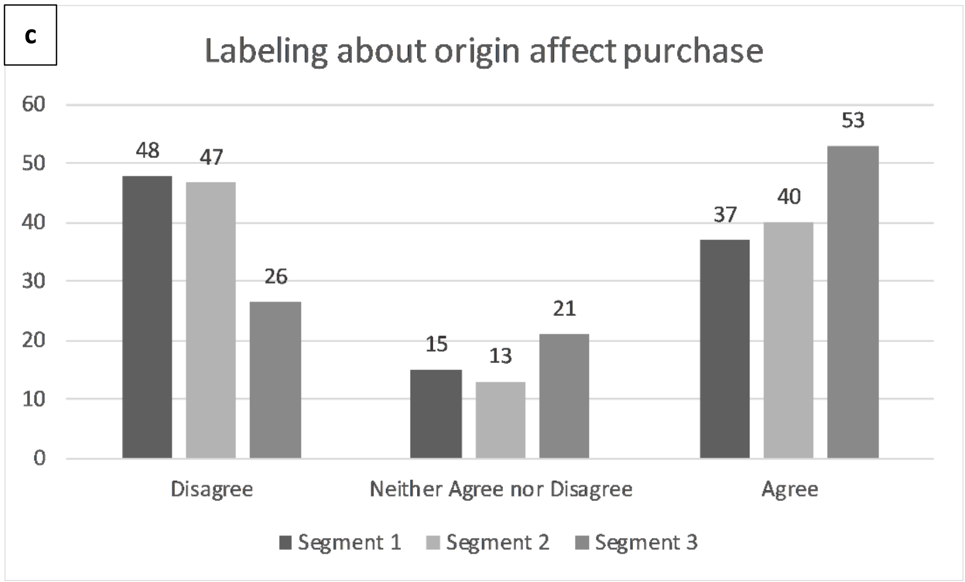

Opinion – Labeling about origin (like Idaho, Colorado, etc.) of potatoes influence my purchase decision♦ (*N* = 95)

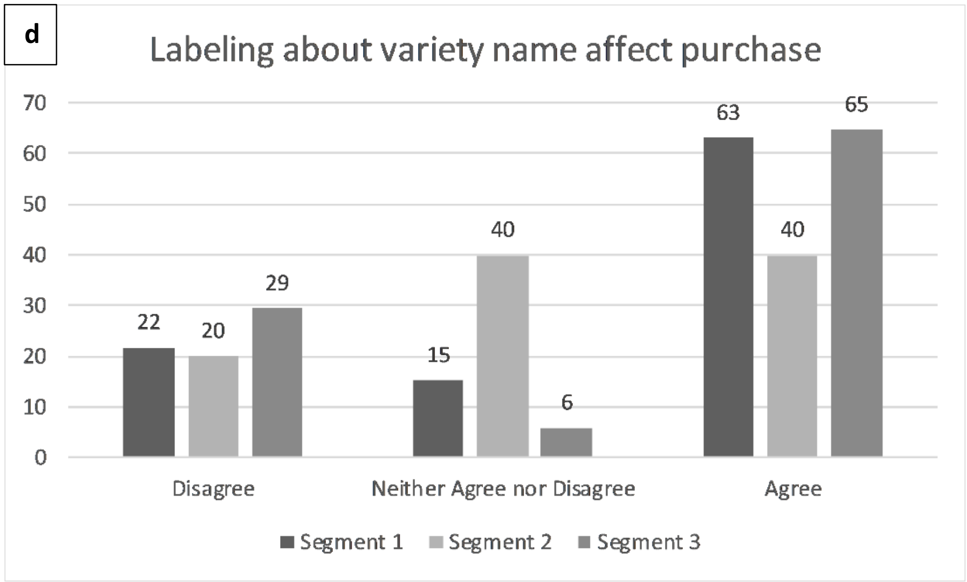

Opinion – Labeling about variety name (like Russet Norkotah, Snowden, etc.) on the package influences my purchase decision (*N* = 95)

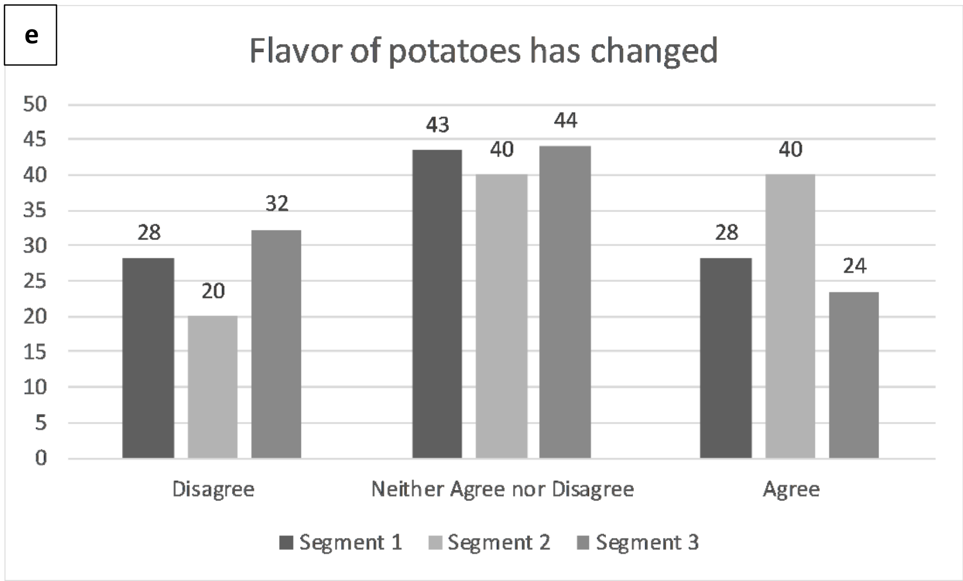

Opinion – I believe that the flavor of potatoes has changed historically♦ (*N* = 95)

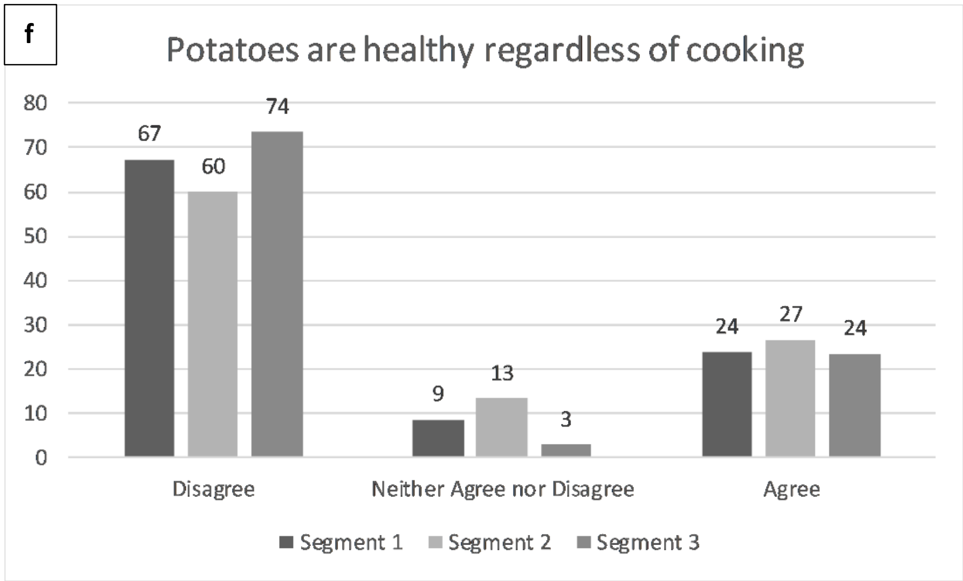

Opinion – Potatoes are healthy regardless of the cooking method♦ (*N* = 95)

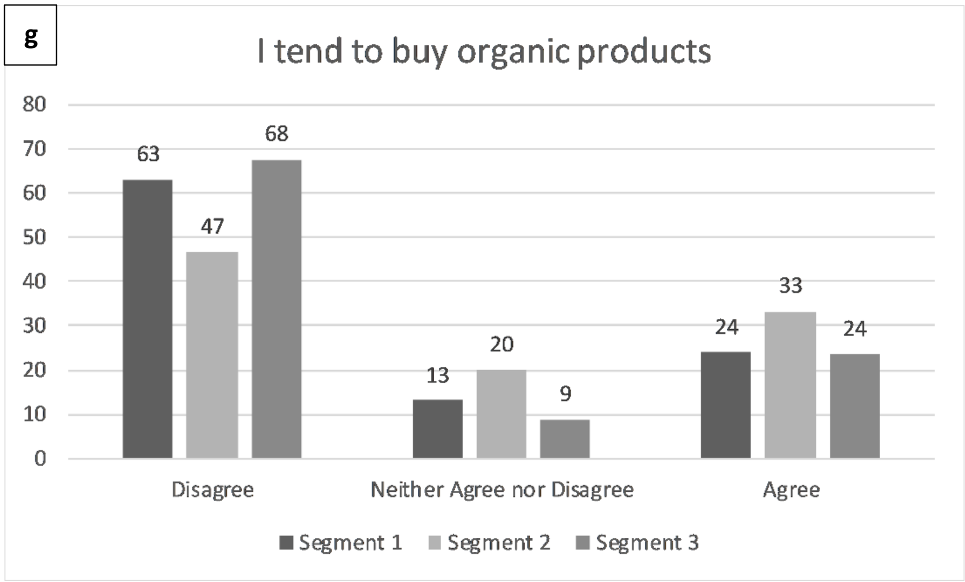

Opinion – I tend to buy organic products/ingredients♦ (*N* = 95)

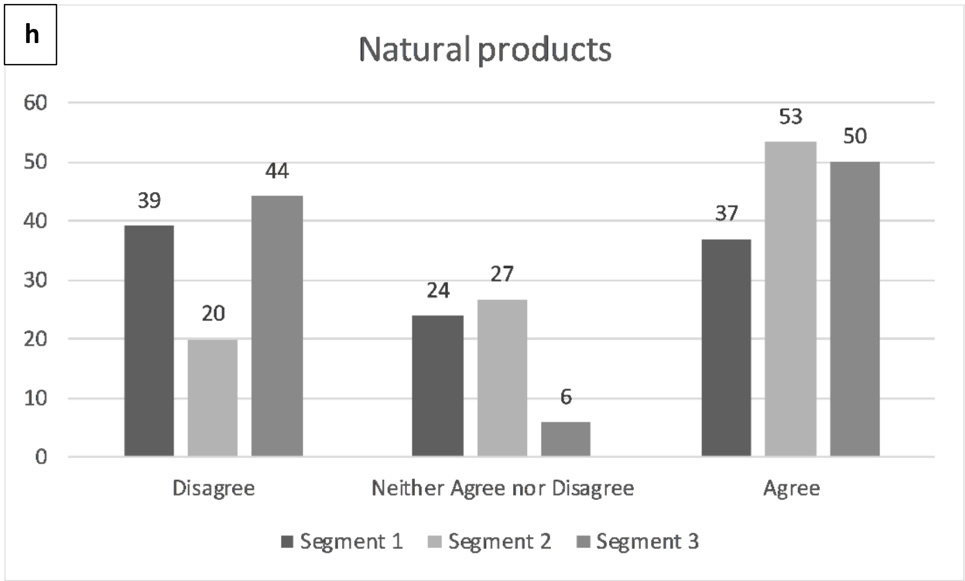

Opinion – I tend to natural products or ingredients♦ (*N* = 95)

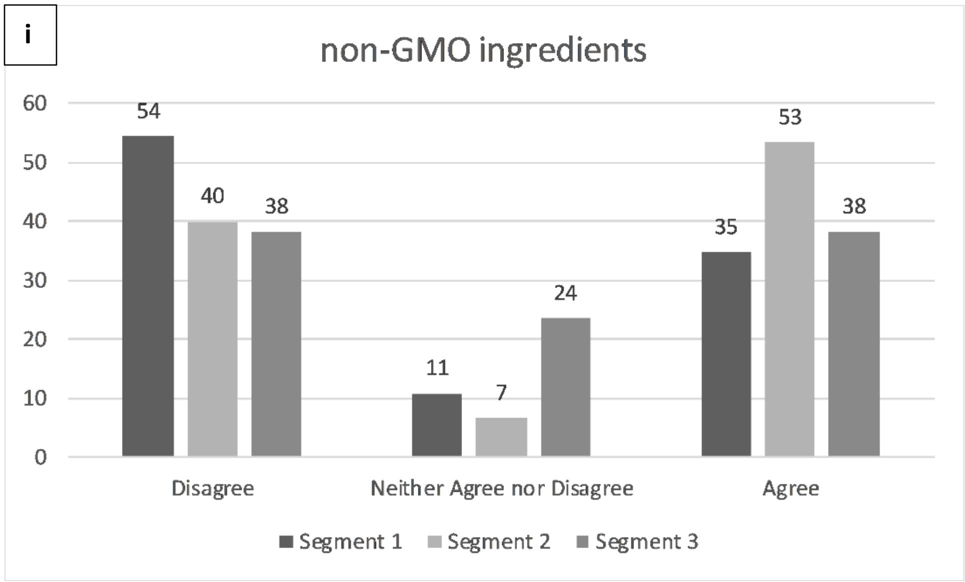

Opinion – I look for non-GMO (Genetically modified organisms) ingredients in the food I eat♦ (N = 95)

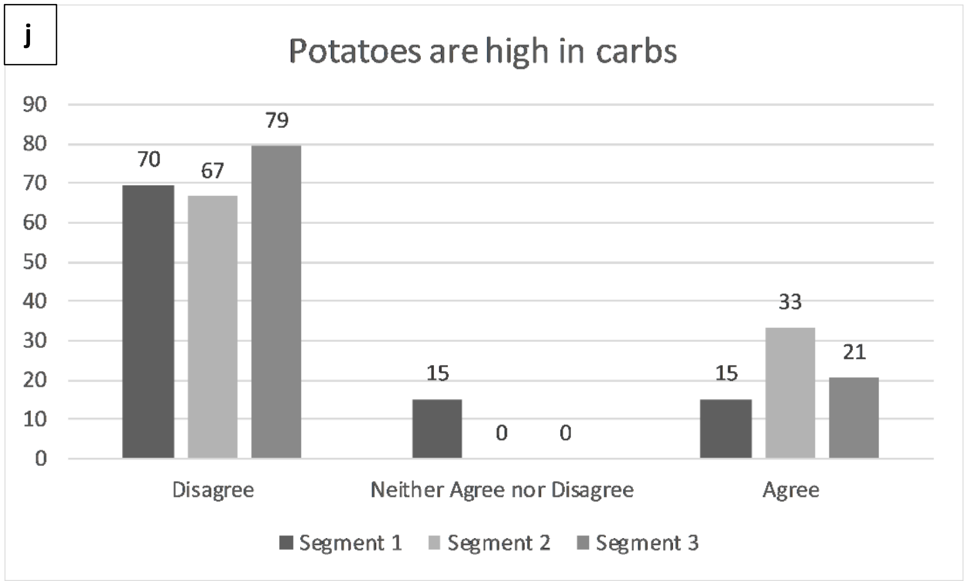

Opinion – I avoid potatoes because they are high in carbohydrates (N = 95)
